# Investigating Saudi university medical students’ English language difficulties: a needs analysis study

**DOI:** 10.3389/fmed.2024.1492031

**Published:** 2025-01-03

**Authors:** Mohammed Alrashed, Muhammad M. M. Abdel Latif

**Affiliations:** ^1^College of Languages and Translation, Imam Mohammad Ibn Saud Islamic University (IMSIU), Riyadh, Saudi Arabia; ^2^Faculty of Graduate Studies of Education, Cairo University, Giza, Egypt

**Keywords:** English for medical purposes, health professional education, medical English, medical students’ English needs, needs analysis

## Abstract

An important dimension in the education of health professionals is developing their English language performance to help them meet academic and professional needs. Though understanding Saudi university medical students’ English language needs is key to helping them perform well in their academic studies and future workplaces, there has been scarce research on these needs. The present study investigated Saudi university medical students’ English language difficulties, the strategies they use for overcoming these difficulties, their evaluation of the English language instruction received at their colleges and perceptions of their English language needs. The study drew upon surveying students’ language needs through using a 25-item questionnaire with four sections assessing the target dimensions. Two hundred and 55 students completed the questionnaire. The students have some difficulties when completing productive language tasks and when processing content explained or written fully in English. Their highest language needs relate to improving medical terminology and English speaking, but their lowest language need concerns listening. A few significant gender-related differences were noted. The English language instruction provided to the students studying medicine could be reformed through giving more attention to improving their productive language skills and helping them communicate fluently using target medical terminology.

## 1 Introduction

English language skills are necessary for medical students and professionals. Since internationally published medical books, articles and journals are written in English, it is necessary to help students studying medicine reach desired English proficiency levels enabling them to meet academic and professional tasks (1). According to Lodhi et al. (2), “as far as medical profession is concerned, doctors need English language during their academic studies as well as in their professional settings” (p. 205). In many international medical contexts, improving language communication is a necessary need for doctors’ successful career. For example, completing an English language assessment successfully is a pre-requisite for non-native English medical professionals to get a job in European Union countries (3). As a result, academic medicine education programs should aim at improving students’ language skills and their ability to perform a variety of language tasks (4). Given that medical professionals should be able to communicate clearly and effectively with patients and their families, and workplace colleagues, in-depth language preparation is needed for medical students (5, 6).

The process of identifying medical students’ language learning priorities and providing them with effective English instruction should start with analyzing their needs. Needs analysis helps in determining students’ real needs and structuring their language curriculum in light of their own communication needs and wants (7–9). Talking about the importance of language needs analysis in medicine education, Kayaoğlu and Dağ Akbaş (10) state that:

This dominance of English in medical accounts paves the way for emergence of a new ESP (English for Specific Purposes) branch as EMP (English for Medical Purposes). The basic insight into this trend is to offer course design, content and materials by being responsive to target language learners’ own agenda. Therefore, it is necessary to find out first what is specifically appropriate, available and applicable for the target situation and target language learners in terms of their needs. In discovering their needs, needs analysis is regarded as an integral part of decision making processes in EMP. Without conducting a needs analysis process, using a medical English course book might not be enough (p. 63).

Not many studies have been conducted on language needs assessment of medical students. Additionally, the few studies available have some limitations related to the linguistics area(s) covered or the context addressed. In an attempt to address this research gap, the present study explored Saudi university medical students’ English language difficulties and needs.

## 2 Literature review

Previous studies have explored medical students’ English language needs from different angles. These studies have also been concerned with different international contexts. Some studies were related to South-East Asian or Western educational settings. For example, Karimnia and Khodashenas (11) conducted a study in Korea about the relationships between medical students’ attitude toward English-medium instruction and their English proficiency levels. Their student participants had diverse needs for performing English language tasks in medical study and for remedial English courses. In Indonesia, Wahyuni (12) used a questionnaire to look at medical students’ academic and professional English needs. Most students in this study viewed improving English listening and speaking skills as their top professional language needs. As for the academic needs, the students rated communicative skills and academic reading strategies as their main priorities. In a mixed-methods study which used a questionnaire, and student and teacher interviews, Reynolds, Zhang and Ding (13) investigated Taiwanese students’ English medical vocabulary learning and difficulties. The students were found to encounter difficulties in learning unfamiliar medical vocabulary due to insufficient contextualized writing practice it, the lack of teacher feedback on their vocabulary use, and inadequate pedagogical communication among faculty members. In a survey study with non-native English-speaking prospective physician applicants with an undergraduate or graduate degree from U.S. accredited higher education institutions, Najmabadi et al. (14) investigated the relationship between self-reported English proficiency levels and the likelihood of physician assistant matriculation. The applicants with low English proficiency levels had a limited potential for physician assistant matriculation. Najmabadi and colleagues conclude that matriculating and training physician applicants with English language concordance are important means of improving medical service outcomes.

Another group of relevant studies have been conducted in Mid-Eastern non-Arab countries. In Iran, Sadeghi et al. (15) examined the correlation between Iranian medical students’ English levels and their scores on medicine courses scores. Their study revealed that the students’ English proficiency significantly correlated with their academic achievement. Two other recent mixed-method studies were also reported about medical students’ language needs in Iran. In Atai and Abbasi’s (16) study, the students and medical faculty members differed in rating the importance of the students’ language needs. The medical students in this study had improvement needs in all language areas, but the English courses they were studying only met their English reading needs. On the other hand, Khalili and Tahririan (17) found that teaching medical English in five Iranian colleges of medicine was hindered by inappropriate materials, students’ low motivation, and heterogeneous English language classes. Their study also indicated that the four language skills were not practiced systematically in the English courses the students were studying.

Other relevant studies in Mid-Eastern non-Arab contexts relate to Turkey, Pakistan and Ethiopia. In Turkey, Kayaoğlu and Dağ Akbaş (10) investigated the academic English needs of medical students through using a questionnaire covering students’ purposes of learning English and skills needs. They found that speaking was the most important skill for students to improve and it was followed by listening, reading and writing. In Pakistan, Lodhi et al. (2) surveyed the English language needs of medical students and in-service doctors. They found a large gap between the required workplace English skills and the realities of English instruction provided to medical students. The respondents in this study called for integrating different English instruction into medical students’ academic programs. On the other hand, Gayessa and Mohammed (18) explored health sciences students’ English needs at an Ethiopian university through using observations and interviews. Compared to reading and grammar, their participant students had more difficulties in writing, medical terminology, speaking and listening.

Some studies have been concerned with Arab university medical students’ English language needs. In Egypt, Sabbour et al. (19) surveyed the views of medical students and faculty members on using English as a medium of instruction. Due to the many English difficulties encountered, the larger number of students in this study preferred to use Arabic as a language of studying medical courses and they depended on translation in their medical study practices. In the United Arab Emirates, Mohamed, Aljadaan and Al-Ani’s survey study (20) revealed that medical students greatly value ESP courses as their major linguistic interest was to develop medical terminology and communicative skills. In Yemen, Farea and Singh (21) used questionnaires and semi-structured interviews to identify medical students’ language needs. They found discrepancies in the students’ and lecturers’ perceived language priorities. Listening and reading were the students’ most important skills, but their lecturers viewed writing and listening as more important. Using a questionnaire, Tayem et al. (22) examined the English language barriers potentially influencing medical students’ achievement at a Bahranian university. Most students in this study thought that English proficiency levels did not influence their academic performance. The study also revealed that Bahranian university students had concerns about their achievement in medical terms and English oral ability.

The relevant studies in the Saudi context are very scarce. One of these scarce studies was reported by Alhamami and Almelhi (23) who used two questionnaires to survey the use of English as a medium of instruction in healthcare majors. They particularly focused on a sample of students studying medicine, dentistry, pharmacy, nursing, and applied medical sciences at a Saudi university. They found some obstacles to using English in teaching healthcare students in Saudi Arabia; students’ poor English skills had a negative effect on their academic achievement, and oral fluency was the students’ major English language weakness. Another study was conducted by Qadeer and Chow (24) who used a student questionnaire and teacher interviews to examine foundation year medical students’ English difficulties and lacks at Saudi university. In this study, the students had more difficulties in English writing and reading as compared to speaking and listening; writing was their most challenging language area.

On the other hand, very little attention has been given to investigating gender-related differences in medical students’ English learning and performance. The studies dealing with this issue are very few. For example, Sojoodizadeh, Ahangari and Sheykhsaran (25) examined Iranian health sciences students’ English language needs and learning expectations. Their questionnaire data showed that the female students had significantly higher general expectations for their English learning than the male students who had higher specific language learning expectations. In the Saudi context, Alshahrani (26) used teacher interviews and student questionnaires to explore how female and male medical students differ in language use and communication styles in online courses. Alshahrani’s study showed gender-related differences in the students’ language use and also in their teachers’ interactions with them in online English classes. In a survey study about medical students’ learning difficulties at Umm Alqura University in Saudi Arabia, the female students in Almoallim et al.’s study (27) rated English language as their first learning difficulty unlike the male students whose first rated difficulty was peer competition. Apart from these rare studies, other published studies tackling the role of gender in medicine education have been concerned with non-linguistic issues such as students’ choice of specialization (28), their learning styles (29), and psychological perceptions (30, 31). Overall, the paucity of research on the relationship between medical students’ gender and their English language learning suggests the dire need for exploring this issue to optimally meet the linguistic needs of female and male students studying medicine.

Given the scarcity of the studies dealing with Saudi medical students’ language needs and the role of gender in these needs, the present study dealt with this issue from a different angle. Specifically, the study investigated medical students’ English language difficulties, the strategies they use for overcoming these difficulties, their evaluation of the English language instruction received at their colleges and perceptions of their English language needs. Additionally, the study also examined the potential gender-related differences in these four dimensions. What distinguishes the present study from previous ones concerned with the Saudi context (23, 24, 26, 27) is its educational setting, scope, and data source. This study focused on Saudi university medical students only. Unlike these previous studies, the present study involved students from more than one Saudi university and it also addressed gender-related differences from a wider perspective. Besides, the needs analysis dimensions covered by the survey tool used in the present study are different. The importance of this study lies in that it could contribute to profiling English language needs of medical students in Saudi Arabia; a context in which English medium instruction university students encounter language difficulties in other university majors (32–34).

In light of the above, the study is guided by the following four research questions:

1. To what extent do female and male Saudi university medical students find difficulties in studying their major in English?
2. Which coping strategies do female and male Saudi university medical students use more or less in overcoming the difficulties in studying their major in English?
3. How do female and male Saudi medical students evaluate the English language instruction and support they have received during their university study?
4. How do female and male Saudi university medical students perceive their English language needs?

As indicated in the introductory section, the present study drew upon needs analysis which can be used as a basis for developing course contents, and meeting health professionals’ language needs, wants, and interests (8). In other words, need analysis is viewed as an integral part of deciding upon language learners’ needs and the way of meeting these needs (11, 35). The data for analyzing learners’ needs can be collected through different sources and from stakeholders studying or working in the target field (36). Since surveys have been regarded as a primary data source for informing language curriculum and program developers about target leaners’ needs (8), the study used a questionnaire to examine the students’ beliefs and reported practices related to the pertinent issues and in turn answer its research questions. The study could provide very important insights into the realities of medical students’ English language learning and their linguistics needs in Saudi Arabia.

## 3 Materials and methods

### 3.1 Context and participants

The participants of this study were medical students studying at public Saudi universities. Prior to starting their medical study, these students attend a foundation year in which they study some preparatory courses in English along with other areas related to their major. The students attending this foundation year come from different pre-university school backgrounds; some students have attended public schools in which they received simple English language instruction, whereas others have been private school students and received intensive English language instruction. The English courses they study in the foundation year vary from one university to another, but these normally cover the four language skills and medical terminology. The language courses generally aim at preparing students for studying medical subjects in English, and for helping them obtain a required score on some standardized international tests such as the IELTS. For these students to start their study at the colleges of medicine, they have to pass the foundation year with a required score on one of the target international tests. After joining their academic medicine program, students do not receive English language instruction at the colleges as their study becomes limited to medical content.

The participants in this study were 225 medical students studying at four public Saudi universities. A hundred and thirty-eight students were males and 117 were females. The 255 students were all Saudis. During the data collection stage, these students were in different academic levels in the first two academic years of their study. All participant students agreed to take part in the study voluntarily. Institutional ethical approval and informed consent from the students were obtained.

### 3.2 The questionnaire

As indicated above, the study used a questionnaire for collecting its data. Instead of using a previously standardized survey, the authors decided to develop their own questionnaire to assess the issues and dimensions peculiar to the present study. According to DeVellis (37), different measures assess unique dimensions of the same general phenomenon. The questionnaire was developed in light of the purpose of the study and the issues it addressed. Prior to developing the questionnaire, the first author visited one college of medicine and conducted three focus group interviews with the students in it to initially collect data about the language-related tasks they perform in their medical study, the coping strategies they resort to when encountering a language problem, and the linguistic areas they were interested in developing. The interview data helped us build the questionnaire in the optimal way possible, and include the most relevant statements in it. Though online meetings and discussion, we developed our agreed-upon conceptualization on the questionnaire format and sections, and the items in each section. Based on this conceptualization, the second author wrote an initial questionnaire draft which was approved by the first author after editing a few statements in it. The questionnaire was originally developed in English, but we translated it into Arabic– the target student population’s native language– to avoid students any potential difficulties in understanding its statements. We asked two expert applied linguists to read the English and Arabic versions of the questionnaire along with our study research questions, and to provide us with their evaluative comments regarding the suitability of the items to the students and to the purpose of the study. The two applied linguists’ views were positive, but we deleted one redundant item and rephrased five statements in the questionnaire in light of their suggestions. Collectively, the average agreement percentage of the applied linguists’ evaluation with the questionnaire draft they read was 93%. Following this stage, we piloted the Arabic questionnaire version with two classes of medical students at a Saudi university to see if they would have difficulties in understanding its statements. The piloting indicted that the students were able to understand all the questionnaire statements with no difficulties.

In its final version, the questionnaire includes four sections with 25 items assessing the four dimensions the study focused on. It starts with a demographic part used for collecting data about the students’ university, gender, nationality, and academic level. The first questionnaire section consists of eight items tapping the respondents’ ratings of the difficulty of some language-related tasks. A 5-point Likert scale (very difficult, difficult, not sure, not difficult, not difficult at all) was used in this section. The second section includes eight statements assessing the strategies the students use for coping with English language difficulties in their medical study. A 5-point Likert scale (always, often, sometimes, seldom, and never) was used in this second section. As for the third and fourth sections, these assessed the evaluation of the English language instruction and support they have received during their medical study (*n* = 4 items), and their reported English language needs for medical study (*n* = 5 items). In both sections, we used a 5-point Likert scale (strongly agree, agree, neutral, disagree, and strongly disagree). Statistical analyses indicated that the 25 questionnaire items had an average Cronbach’s alpha reliability coefficient of 0.78, which is regarded a good and acceptable reliability coefficient. See the full form of the questionnaire in Appendix 1.

### 3.3 Data collection and analysis procedures

The students’ responses to the questionnaire were collected over 5 weeks. We circulated the Arabic questionnaire version to colleague faculty members through emails and WhatsApp, and they in turn passed it to their students. We kept inviting the student population to complete the questionnaire until the end of week five. Following the data collection, we analyzed the data through running the descriptive analyses of the female and male students’ responses to the questionnaire statements. As for inferential analyses, we used the one-way ANOVA to analyze the differences between the female and male students’ responses to the items.

## 4 Results of the study

The results of the study are given in the following subsections. As will be noted, these results are presented in light of the research questions.

### 4.1 The students’ ratings of the difficulty of performing language tasks

Table 1 shows that the means and standard deviations along with the one-way ANOVA analysis of the students’ responses to the questionnaire items tapping their ratings of the difficulty of performing some language tasks. As noted, the statements are arranged depending on the overall mean responses (from the least difficult to the most difficult). The students’ ratings of the difficulty of the given tasks vary from one task to another, though such variance is not large. The two easiest language tasks for both the students are understanding a lecture and content partially explained or written in English (overall mean = 2.3 and 2.4, respectively), but their task difficulty increases if the content is fully explained or written in English (overall mean = 3.1 for both tasks). Unlike receptive skill tasks (i.e., those related to listening and reading), the students find it a bit more difficult to deal with productive tasks such as using English in answering oral tests and exams or a term assignment in writing English (overall mean = 3.1 for both tasks), compare statements 7 and 8 in the table to statements 1 and 2.

**Table 1:** The students’ ratings of the difficulty of language-related tasks in their medical study.

To what extent do you find the following situations difficult in your medical study?	Males		Females		Overall mean	Mean square	*F*	Sig.
	*M*	SD	*M*	SD				
1. Understanding a lecture partially explained in English	2.2	1.1	2.4	1	2.3	1.9	1.7	0.1860
2. Understanding subject content partially written in English	2.3	1.05	2.4	1	2.4	0.7	0.70	0.3970
3. Using English in answering written tests/exam questions	3	1.4	3	1.2	3	0.1	0.1	0.8850
4. Using English in raising questions and discussing subject content during lectures	3	1.4	3	1.2	3	0.1	0.00	0.9350
5. Understanding a lecture fully explained in English	2.9	1.2	3.2	1.3	3.1	5.3	3.3	0.0690
6. Understanding subject content fully written in English	2.9	1.3	3.2	1.2	3.1	3.2	2	0.1560
7. Using English in answering oral tests/exam questions	2.9	1.2	3.2	1.2	3.1	5.1	3.6	0.0580
8. Writing a term assignment in English	3	1.2	3.1	1.2	3.1	0.2	0.1	0.7680

Collectively, the overall means of both female and male students on these tasks imply that they have some difficulty in performing particular language tasks in their medical study. Specifically, they encounter difficulties in using English for performing academic writing and speaking tasks (statements 3, 4, 7 and 8) and also in understanding academic medicine materials fully explained or written in English (statements 5 and 6). The students’ responses to statements 5 and 6 indicate they expect to have some translation when listening to lectures or reading their academic materials.

The table also shows that the male students find it a bit more difficult to perform some tasks as compared to their female peers. This can be noted in the female and male students’ responses to statements 1, 2, 5, 6, 7 and 8 in the table. However, the one-way ANOVA analyses indicate that all these differences are non-significant with the exception of statement 7 (*p* = 0.05). These results mean that compared to the females, the male students find it more difficult to use English in answering oral tests/exam questions; apart from this, there are hardly any differences between the females and males in performing the other tasks listed in the table.

### 4.2 The students’ coping strategies with English language difficulties

Table 2 gives the statistical analyses of the students’ responses to the questionnaire items assessing the strategies they use for coping with English language difficulties. These strategies are arranged in the table from the most to the least frequently used ones. As noted, the female and male students try to solve their language problems more by exerting efforts in improving their English than by relying on private English tutoring (overall means = 4 and 2.9, respectively). The students’ answers to statements 2, 4, 5, 7 and 8 indicate they depend on the translation of the medical content being learned.

**Table 2:** Medical students’ reported strategies for coping with English language difficulties.

When having a difficulty in English in my medical study, I……	Females		Males		Overall mean	Mean square	*F*	Sig.
	*M*	SD	*M*	SD				
1. Try to improve my level in English.	4.4	1	4	1.2	4.2	10.2	8.1	0.005
2. Translate a part of the content in Arabic.	3.3	1.23	3.7	1.17	3.5	9.5	6.7	0.010
3. Watch an online English video about the topic.	3.5	1.15	3.4	1.13	3.5	0.4	0.3	0.593
4. Watch an online Arabic video about the topic.	2.7	1.17	3.3	1.37	3.1	16.2	9.9	0.002
5. Read an Arabic content about the topic.	2.8	1.3	3	1.3	2.9	3.9	2.3	0.127
6. Rely on private English tutoring.	2.8	1.36	2.9	1.40	2.9	0.2	0.1	0.807
7. Ask the lecturer to explain the content in Arabic.	2.5	1.33	3	1.25	2.8	19.9	12.1	0.001
8. Translate the whole content in Arabic.	2.7	1.43	2.8	1.36	2.8	1.4	0.8	0.383

The students’ responses to statements 3 and 4 also imply they depend on visuals or videos in solving the language problems encountered. As noted, the students rely more on viewing online English videos than Arabic ones (overall means = 3.5 and 3.1, respectively). This may be ascribed to the fact that there are more medical online video materials available in English than in Arabic. Overall, the descriptive data indicate that the students solve their English language problems mostly through depending on visuals and partial or full translation of the English content; but they tend to depend more on partial rather than full translation. The students also try to develop their language levels as a way for solving their language problems. Meanwhile, they do not frequently seek language support either from their content teachers or private English tutors.

With regard to gender-related differences, the data shows that the females are more active in their English language development, and that the males resort more to translanguaging, i.e., translating English content into Arabia or using translated medical learning materials. As noted in the table, the differences between the responses of the female and male students are significant in four statements (*p* = 0.005, 0.010, 0.002, 0.001 for statements 1, 2, 4 and 7, respectively), and non-significant in the other 4 ones. These significant differences particularly mean that unlike the females who try to overcome academic language problems through improving their English, the male students depend more on translation in their study of medicine.

### 4.3 The students’ evaluation of the English language instruction and support received

Table 3 provides the statistical analyses of the students’ responses to the items related to their evaluation of the English language instruction and support they have received during their medical study. As can be noted, the students generally value the efforts made by their medical faculty members and the language teachers who have taught them in the foundation year (overall means = 4 and 3.9, respectively). On the other hand, they rate their university English courses and their contributions in improving their English in a slightly lower way (overall mean = 3.7). Their responses to statement 2 mean they believe there is a need for modifying the English courses at their colleges (overall mean = 3.5). Overall, these responses indicate that while the students appreciate the efforts made by their content and language teachers, they generally hold the belief that the language courses they studied are to be modified.

**Table 3:** The students’ evaluation of the English language instruction and support they have received during their medical study.

To what extent do you agree or disagree with the following?	Females		Males		Overall mean	Mean square	*F*	Sig.
	*M*	SD	*M*	SD				
1. The university English courses helped in improving my language level in a way meeting study requirements.	3.6	1.2	3.8	1	3.7	3.3	2.7	0.101
2. English language courses at the College need to be modified.	3.4	1.3	3.6	1.3	3.5	2.592	1.5	0.212
3. Medical faculty members try to help us improve our English level.	4.2	1.1	3.9	1.1	4	3.003	2.3	0.127
4. The College has a group of language teachers who have helped us meet the English requirements.	3.9	1.18	3.9	1.12	3.9	0.179	0.1	0.713

As for gender-related differences, the females and males reported similar responses to the statements about the evaluation of the English language instruction and support. The largest difference noted in the table is in the students’ response to statement 3 where the females rated the efforts made by medical faculty members in improving their English in a higher way than the males (overall means = 4.2 versus 3.9, respectively). However, these differences are non-significant, perhaps due to the similarity of the institutional English language instruction and support services provided to both female and male medical students in the Saudi context. Thus, they rated these services in a similar way.

### 4.4 The students’ English language needs in medical study

The statistical analyses related to the students’ reported English language needs in their study of medicine are given in Table 4. These language needs are arranged in the table in terms of their frequencies (from the highest needed to the least needed areas). As the table explains, the students’ highest language needs concern improving their performance in medical terminology and English speaking (overall means = 4 for both areas). It is likely that the students pay more attention to developing their medical terminology knowledge and pronunciation and oral English, and regard them as requirements for being a successful future doctor. It seems that what matters for medical students in the Saudi context is to orally communicate well and use medical terms properly. Perhaps they expect this will give them a sense of confidence and competence in future clinical situations and patient-doctor interactions. Conversely, the students’ lowest language need relates to listening (overall mean = 3.7). As for writing and reading, these are rated slightly lower than medical terminology and speaking and higher than listening (overall means = 3.9). Overall, the students feel they are in need for developing all the English language areas given in the table. Their reported language needs are generally consistent with their ratings of the English difficulties (see subsection 4.1), and emphasize their need for more support in improving productive language skills.

**Table 4:** The students’ reported English language needs for medical study.

To what extent do you agree or disagree with the following?	Males		Females		Overall mean	Mean square	*F*	Sig.
	*M*	SD	*M*	SD				
1. I need to improve my performance in medical terminology.	4	0.9	4.1	1.2	4	0.1	0.1	0.889
2. I need to improve my English speaking.	4.1	0.9	3.8	1.1	4	4.3	3.8	0.050
3. I need to improve my English writing.	3.7	1.1	4.1	1.1	3.9	10	8.4	0.004
4. I need to improve my English reading.	3.9	1.1	3.9	1.1	3.9	0.1	0.1	0.807
5. I need to improve my English listening.	3.9	1.1	3.8	1.2	3.7	0.5	0.4	0.546

On the other hand, gender-related differences are hardly noted in the students’ responses to statements 1, 4 and 5, but significant differences are found only in the responses to statements 2 and 3 where the females reported a higher need for improving their English speaking (means = 3.8 versus 4.4, respectively, *p* = 0.05) and the males reported a higher need for improving their English writing (means = 4.1 versus 3.7, respectively, *p* = 0.004). These significant differences mean that the females need to develop a better English speaking level while the males may have had less engagement in practicing English writing in previous academic stages or years.

## 5 Discussion

This study has revealed important results about Saudi university medical students’ English language difficulties and needs. Regarding the students’ English difficulties, they have some difficulties when completing productive language tasks (i.e., those related to speaking and writing), and when processing content explained or written fully in English. Thus, the study supports previous research findings that Saudi university medical students encounter multiple English difficulties in their academic study (23, 24, 27). As for the students’ strategies for coping with language difficulties, they overcome their English difficulties mainly through watching online videos and resorting to partial or full translation of the English content. Like their peers in Egypt (19, 38), most medical students in Saudi Arabia depend on translation as a main solution for overcoming their academic English difficulties.

On the other hand, the participant students are generally satisfied with the role played by medical faculty members and language instructors in developing their English knowledge and performance, but they have a lower satisfaction level with the English courses studied at their colleges. This case of medical students’ dissatisfaction with English courses is similar to the one found in Iran (16, 17) but is dissimilar to the Taiwanese case where students did not complain about the learning materials but about the lack of teacher feedback on medical vocabulary use and inadequate pedagogical communication (13).

With regard to the students’ English language skill development needs, the students believe they are in need for developing their skills in all language areas. This case of medical students’ need for improving multiple English skills was also found in other international contexts (16). Meanwhile, the students’ highest language needs relate to improving their medical terminology and English speaking while their lowest language need is listening. These results confirm the context-specific nature of medical students’ language needs. In other words, such linguistic needs may vary from one medicine education environment to another. The present results partially concur with some previous research findings. For example, they are consistent with those studies emphasizing medical students’ dire need for developing their speaking fluency and knowledge of medical terms (18, 20, 22, 23). Contrarily, these results are not in line with previous research findings that students in other medicine education settings have different language development priorities such as English listening and speaking skills in Turkey and Indonesia (10, 12, respectively), or listening and reading in Yemen (21). These differences may be attributed to the different contextual factors influencing medical students’ English learning experiences.

As for the relationship between the students’ gender and their English learning experiences, the results generally indicate that the males encounter more difficulties in some language performance dimensions. There were some mean differences in the responses of the females and males to many statements, but a few number of these differences are significant. Compared to the females, the male students have more difficulties in using English in answering oral tests/exam questions. Likewise, the male students reported trying to overcome their academic language problems through translation, but their female peers reported trying to overcome such problems through regularly improving their English. On the other hand, there are no gender-related significant differences in the students’ evaluation of the English language instruction they received during their university study; a finding that could be ascribed to the similarity of this instruction in both female and male campuses. Finally, the females reported a significantly higher need for developing their English speaking while the males reported a higher need for developing their English writing, but there are no gender-related differences in the reported needs for developing listening, reading or medical terminology. Collectively, these results confirm previous research findings (25, 26) that gender-related differences exist in medical students’ English and performance, but they seem to contradict Almoallim et al.’s study (27) in which the female students rated English language as their first medicine learning difficulty. Overall, the present study along with previous relevant research suggest that female and male medical students have some differences in their academic English learning and performance and improvement needs.

## 6 Conclusion

Supported by previous relevant research findings in the Saudi context (23, 24, 26, 27), the present study provided further evidence emphasizing the need for restructuring the English language instruction delivered to Saudi university medical students. Productive language skills and tasks in particular should be prioritized in future academic English programs. Likewise, medical terminology is another top priority area Saudi university students need to improve. Attention should be particularly given to helping students communicate fluently in oral and written English using the target medical terminology. English language support should be available for medical students at Saudi universities as they progress to upper academic levels. This can be accomplished through establishing an English language unit responsible for in-sessional support of students’ language needs. Additionally, gender-related differences in medical students’ English learning needs and expectations are to be considered. Needless to say, medical English teacher training will be key to bringing about all the needed reforms in medical students’ English language instruction and support at Saudi universities. There is also a dire need for the active participation of stakeholders managing Saudi medical student education programs in implementing these English instruction reforms.

This study is not without its limitations. Future research is needed for reaching a clearer picture about Saudi medical students’ English language needs. Such future research attempts could combine different data sources for triangulation purposes, or collect data from other stakeholders about students’ language needs. Gender-related differences in medical students’ English needs is another area worthing much future research. Moreover, further research is needed in other international medicine education environments. All these research attempts could contribute to developing appropriate medical English instruction practices.

## Data Availability

The data of this study is not publicly available to protect the participants’ privacy. The data shall be available upon request to the corresponding author on maalrashed@imamu.edu.sa.
