# Supplementary material for: Investigating Saudi university medical students’ English language difficulties: a needs analysis study
Source: Front Med (Lausanne). 2025 Jan 3;11:1492031. doi: 10.3389/fmed.2024.1492031 (PMC11739117; doi:10.3389/fmed.2024.1492031)
Supplement: Supplementary file 1 [file Table_1.pdf]

Appendix (1). A questionnaire about medical students' English language difficulties

Dear student,

This questionnaire aims at exploring your English language difficulties and improvement needs. Please note that there are no correct or wrong answers to the statements. Therefore, please try to give as honest and realistic answers as possible. All the data collected will be kept confidential and will not be shared with anyone apart from the research team members. Your participation is completely anonymous and voluntarily. Please note that completing this questionnaire means that you accept to participate voluntarily in the study.

Thank you for your cooperation.

The researchers

University

Gender

**Female**		**Male**	

Nationality

Academic level

In the following parts, please tick as appropriate.

Section 1

To what extent do you find the following situations difficult in your medical study?	Very difficult	Difficult	Not sure	Not difficult	Not difficult at all
1. Understanding a lecture partially explained in English					
2. Understanding subject content partially written in English					
3. Understanding a lecture fully explained in English					
4. Understanding subject content fully written in English					
5. Using English in raising questions and discussing subject content during lectures					
6. Using English in answering written tests/exam questions					
7. Using English in answering oral tests/exam questions					
8. Writing a term assignment in English					

Section 2

**When having a difficulty in English in my medical study, I…..**	**Always**	**Often**	**Sometimes**	**Seldom**	**Never**
1. Watch an online English video about the topic.					
2. Watch an online Arabic video about the topic.					
3. Read an Arabic content about the topic.					
4. Translate a part of the content in Arabic.					
5. Translate the whole content in Arabic.					
6. Ask the lecturer to explain the content in Arabic.					
7. Try to improve my level in English.					
8. Rely on private English tutoring.					

Section 3

**To what extent do you agree or disagree with the following statements?**	**Strongly agree**	**Agree**	**Neutral**	**Disagree**	**Strongly disagree**
1. The university English courses helped in improving my language level in a way meeting study requirements.					
2. English language courses at the College need to be modified.					
3. Medical faculty members try to help us improve our English level.					
4. The College has a group of language teachers who have helped us meet the English requirements.					

Section 4

**To what extent do you agree or disagree with the following statements?**	**Strongly agree**	**Agree**	**Neutral**	**Disagree**	**Strongly disagree**
1. I need to improve my English listening.					
2. I need to improve my English reading.					
3. I need to improve my English speaking.					
4. I need to improve my English writing.					
5. I need to improve my performance in medical terminology.					
